# Supplementary material for: The PECAn image and statistical analysis pipeline identifies Minute cell competition genes and features
Source: Nat Commun. 2023 May 10;14:2686. doi: 10.1038/s41467-023-38287-x (PMC10172353; doi:10.1038/s41467-023-38287-x)
Supplement: Supplementary file 1 — Supplementary Information [file 41467_2023_38287_MOESM1_ESM.pdf]

The PECAn image and statistical analysis pipeline identifies Minute cell competition genes and features

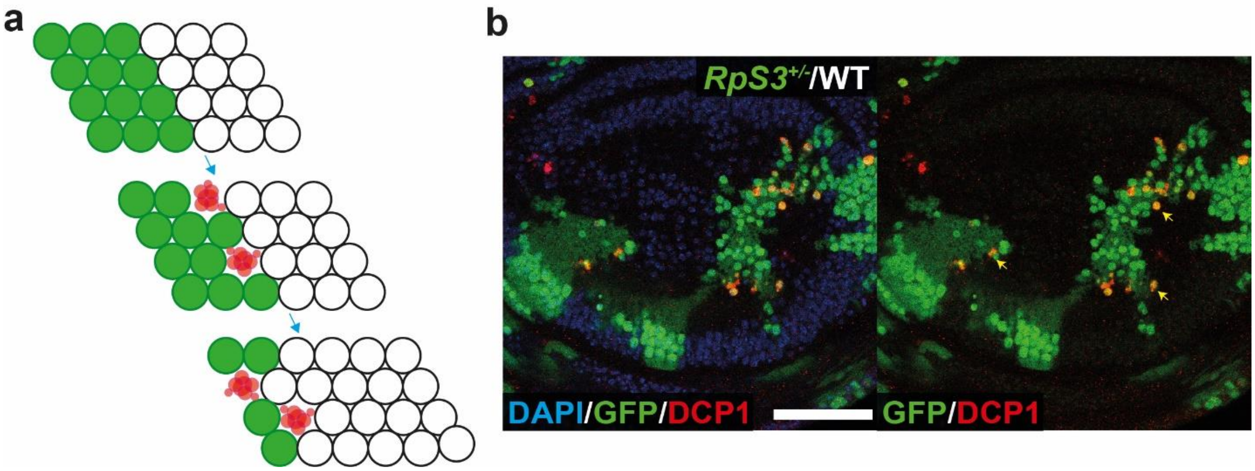

Supplementary Fig. 1: Minute cells are apoptotically eliminated by proximal wildtype cells in mosaic tissues. (**a**) Schematic representation of Minute cell competition. Cells carrying heterozygous mutations in ribosomal protein genes (green) undergo cell competition when confronted with wild-type cells (white). Minute 'loser' cells closest to wildtype cells undergo apoptosis (red). Wildtype 'winner' cells proliferate and fill the vacated space. Over time, the winner population expands, and the loser population contracts. (**b**) An example of Minute cell competition in a wandering third instar *Drosophila* wing disc. Cells heterozygous for *RpS3* (green), a Minute mutation, undergo apoptotic cell death (red, yellow arrowheads) when adjacent to wild-type cells (unlabelled). Scale bar corresponds to 50 µM.

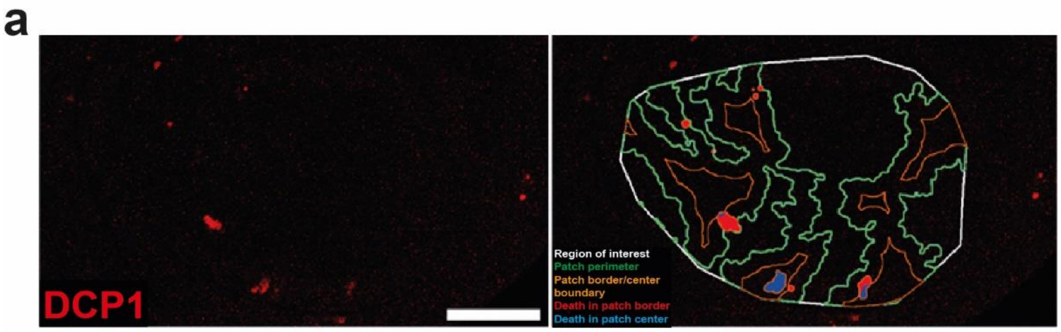

Supplementary Fig. 2: Representative imagej/FIJI PECAn outputs showing cell death segmentation. (Left) The raw cell death channel used for segmentation, along with a 50µm scale bar. This image reflects a competing wing disc stained with anti-Dcp-1 (red). (Right) Overlay of parameters measured by the macro on the raw cell death channel. The white line demarcates the outline of the pouch region. The cell patch perimeter is shown in green, and the patch border/patch centre boundary is in orange. The cell death signal is automatically assigned as occurring in the border region, shown in red, or as occurring in the patch centre, shown in blue.

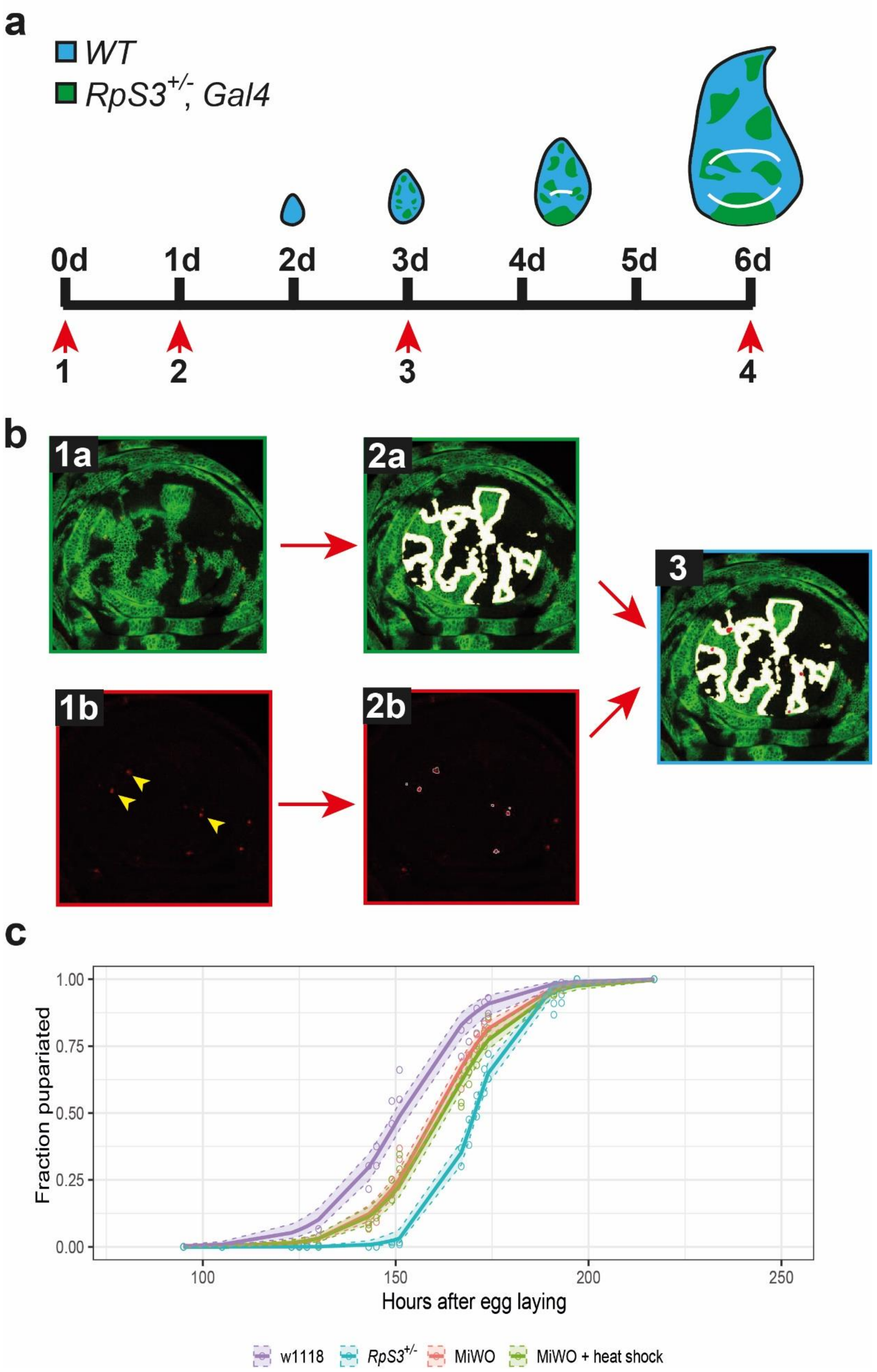

a
WT
RpS3+/-; Gal4
0d
1d
2d
3d
4d
5d
6d
1
2
3
4
b
1a
2a
3
1b
2b
c
Fraction pupariated
1.00
0.75
0.50
0.25
0.00
100
150
200
250
Hours after egg laying
w1118
RpS3+/-
MiWO
MiWO + heat shock

Supplementary Fig. 3: Analysis of Minute cell competition. (**a**) Schematic showing experimental procedure and our strategy for induction of somatic *RpS3*$^{+/-}$ cells, using a genetic tool described in [14]. Larvae of the *hs-FLP, UAS-CD8-GFP/+;; RpS3[Plac92], act>RpS3>Gal4/*+ genotype carry a heterozygous mutation in *RpS3*, and a rescuing transgene driven by an actin promoter, flanked by FRT sites (shown in blue). On day three after egg laying, larvae are heat-shocked to drive transient expression of the FLP recombinase transgene, which is under control of the hsp70 promoter. FLP-induced FRT recombination, results in the sporadic excision of the *RpS3* rescuing transgene, as well as a stop codon. This generates *RpS3*$^{+/-}$ cells expressing Gal4 (under the actin promoter), which in turn induces expression of a UAS-driven GFP (shown in green). Over subsequent days, *RpS3*$^{+/-}$ cells undergo competitive elimination by their wildtype winner neighbours. (**b**) Strategy for quantitative analysis of cell competition. In order to assess cell competition-induced cell death, information is required about both the loser patches (GFP-positive (green), top) and levels of cell death (Dcp-1-positive (red), bottom). First, the region of the patches within two cell diameters of the winners (white overlay in 2a and 3) and the patch centre (the region within the patches further than two cell diameters from the winners), where loser cells are shielded from cell competition, are identified. Then, the numbers of dying cells in the border and centre regions are counted. This information is then combined to obtain densities of apoptotic events in the patch border versus the patch centre. (**c**) Plot showing the cumulative fraction of larvae pupariated on the y-axis and hours after egg laying on the x-axis for wild-type (w^{1118}) larvae (purple), *RpS3*$^{+/-}$ larvae (blue), MiWO larvae which had not been heat shocked (orange), and MiWO larvae which had been heat shocked for 25 minutes (green). Data (dots) were fitted to a sigmoid curve;

the shaded area represents 99% confidence intervals for the fit. Each condition consists of at least five repeats of $n > 40$ larvae. Source data are provided as a Source Data file.

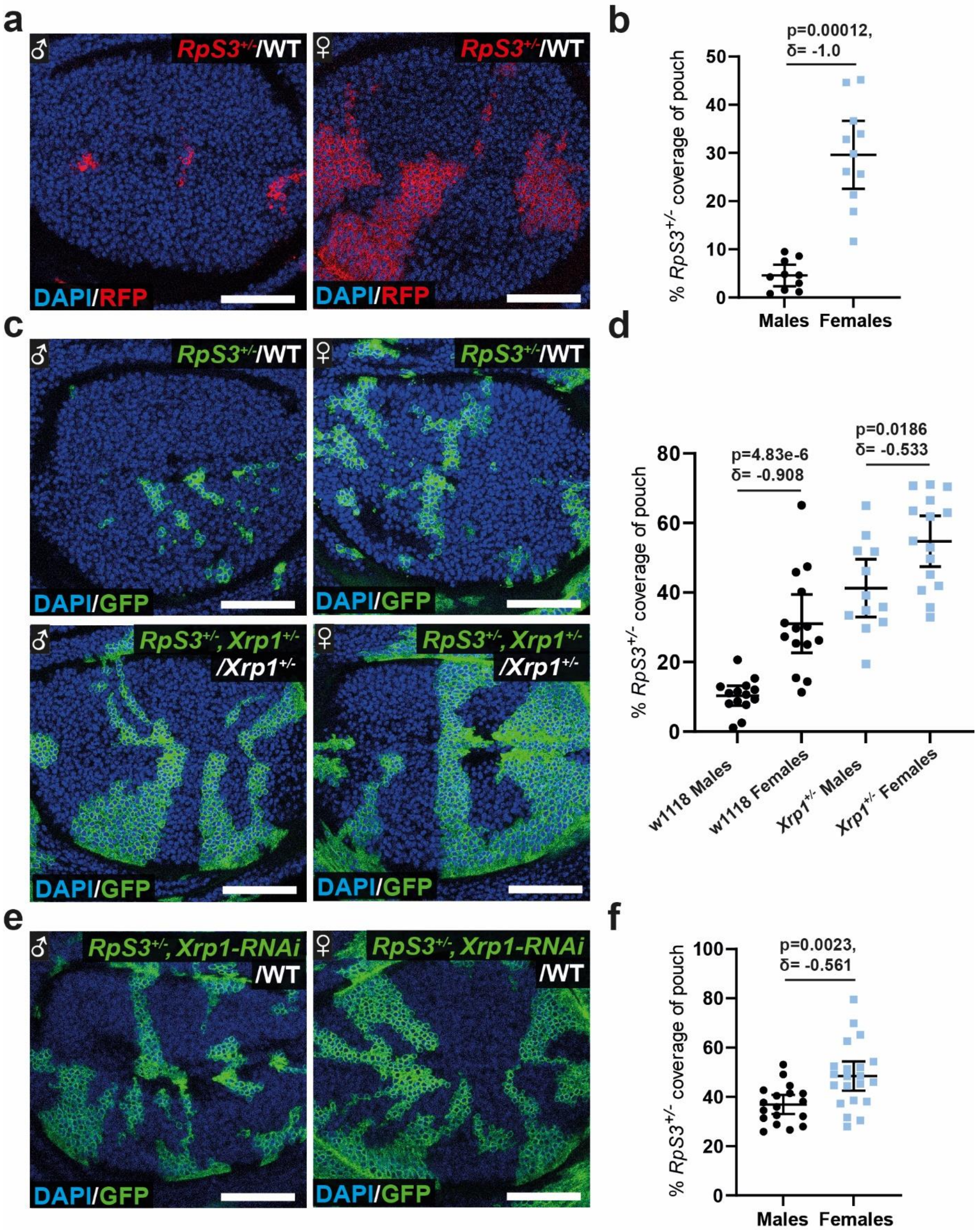
a
♂
RpS3+/-/WT
♀
RpS3+/-/WT
DAPI/RFP
DAPI/RFP
b
p=0.00012,
δ= -1.0
% RpS3+/- coverage of pouch
0
10
20
30
40
50
Males
Females
c
♂
RpS3+/-/WT
♀
RpS3+/-/WT
DAPI/GFP
DAPI/GFP
♂
RpS3+/-, Xrp1+/-
/Xrp1+/-
♀
RpS3+/-, Xrp1+/-
/Xrp1+/-
DAPI/GFP
DAPI/GFP
d
p=4.83e-6
δ= -0.908
p=0.0186
δ= -0.533
% RpS3+/- coverage of pouch
0
20
40
60
80
w1118 Males
w1118 Females
Xrp1+/- Males
Xrp1+/- Females
e
♂
RpS3+/-; Xrp1-RNAi
/WT
♀
RpS3+/-; Xrp1-RNAi
/WT
DAPI/GFP
DAPI/GFP
f
p=0.0023,
δ= -0.561
% RpS3+/- coverage of pouch
0
20
40
60
80
100
Males
Females

Supplementary Fig. 4: Male/female sexual dimorphism is observed with a flippase carried on an autosome and in the context of Xrp1 knockdown. (**a**) Representative images of wing discs from male (left) or female (right) larvae containing *RpS3*$^{+/-}$ losers (red) and wildtype winners (unlabelled) and stained for DAPI. Patches were induced using a *hs-FLP* construct carried on the second chromosome, an autosome. (**b**) Quantification of loser pouch coverage of wing discs as in (**a**). Statistics reflect two-sided Wilcoxon-Mann-Whitney U test with Cliff's δ effect size. Measure of center and error bars are shown as mean and 95% CI, respectively. Biologically independent samples per replicate are as follows: replicate 1: n_{Males}=10, $n_{Females}$=11; replicate 2: n_{Males}=6, $n_{Females}$=8; replicate 3: n_{Males}=7, $n_{Females}$=8. (**c**) Representative images of wing discs from male (left column) or female (right column) larvae containing *RpS3*$^{+/-}$ losers (green) competing against wildtype winners (unlabelled) in a wild type (*w*^{1118}) background (top row) or in a background heterozygous mutant for *Xrp1* (bottom row). Wing discs are stained for DAPI (blue). (**d**) Quantification of loser pouch coverage of wing discs as in (**c**). Statistics reflect two-sided Wilcoxon-Mann-Whitney U test without adjustment for multiple comparisons with Cliff's δ effect size. Measure of center and error bars are shown as mean and 95% CI, respectively. Biologically independent samples per replicate are as follows: $n_{w1118Males}$=14, $n_{w1118Females}$=14, $n_{MiWOMales}$=12, $n_{MiWOFemales}$=15. (**e**) Representative images of wing discs from male (left) or female (right) larvae with wildtype winners (unlabelled) competing against *RpS3*$^{+/-}$ losers expressing *Xrp1-RNAi* (green). Wing discs are stained for DAPI (blue). (**f**) Quantification of loser coverage of wing discs as in (**e**). Statistics reflect two-sided Wilcoxon-Mann-Whitney U test with Cliff's δ effect size. Measure of center and error bars are shown as mean and 95% CI, respectively. Biologically independent samples per

replicate are as follows: replicate 1: n_{Males}=18, $n_{Females}$=21; replicate 2: n_{Males}=18, $n_{Females}$=19. Scale bars correspond to 50 µM. Source data are provided as a Source Data file. ♀ symbol denotes females, ♂ denotes males.

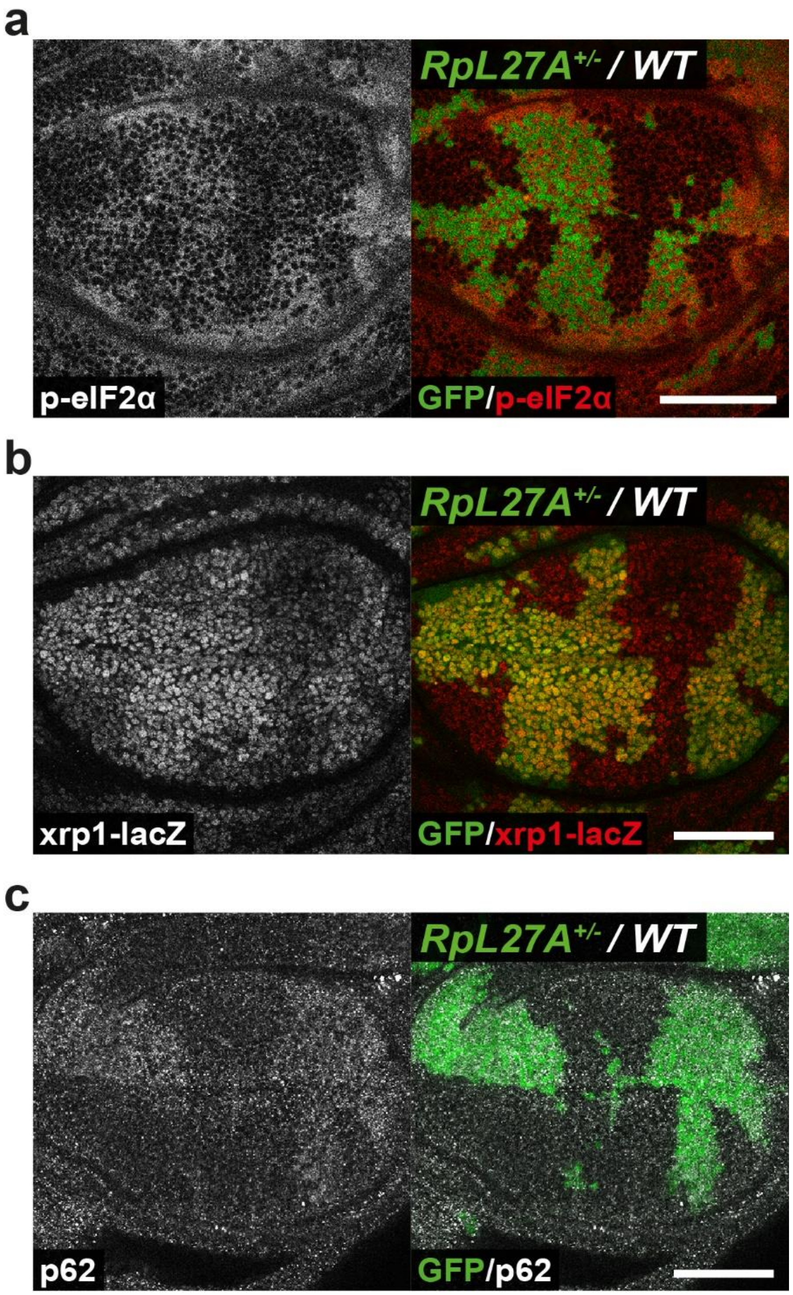

Supplementary Fig. 5: *RpL27A*$^{+/-}$ loser cells display increased p-eIF2α, increased *xrp1* expression and p62 accumulation. (**a-c**) Mosaic wing discs harbouring wildtype cells (unlabelled) and *RpL27A*$^{+/-}$ cells (green) immuno-stained for either p-eIF2α (grey, **a**), beta-galactosidase to detect *xrp1-lacZ* reporter expression (grey, **b**), or p62 (grey, **c**). Scale bars correspond to 50 µM.

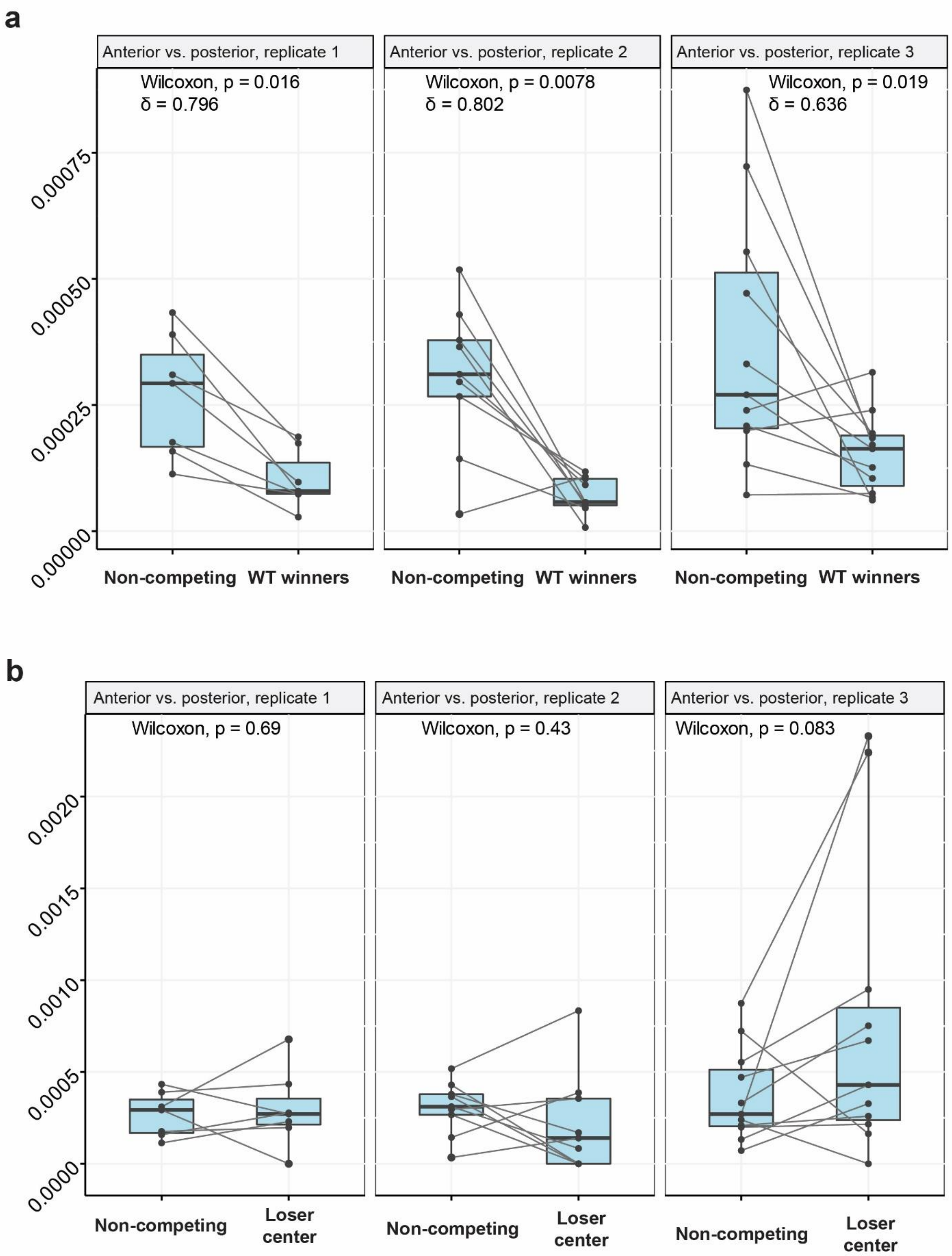
a
Anterior vs. posterior, replicate 1
Wilcoxon, p = 0.016
δ = 0.796
Anterior vs. posterior, replicate 2
Wilcoxon, p = 0.0078
δ = 0.802
Anterior vs. posterior, replicate 3
Wilcoxon, p = 0.019
δ = 0.636
0.00000
0.00025
0.00050
0.00075
Non-competing
WT winners
b
Anterior vs. posterior, replicate 1
Wilcoxon, p = 0.69
Anterior vs. posterior, replicate 2
Wilcoxon, p = 0.43
Anterior vs. posterior, replicate 3
Wilcoxon, p = 0.083
0.0000
0.0005
0.0010
0.0015
0.0020
Non-competing
Loser center

Supplementary Fig. 6: Non-competing *RpS3*$^{+/-}$ cells exhibit higher levels of cell death than competing winners and an equivalent level of cell death to loser cells in the patch centre region. (**a**-**b**) Quantifications of samples as shown in Figure 5. Output graphs and statistical tests generated by PECAn showing the density of Dcp-1-positive cells in the non-competing *RpS3*$^{+/-}$ posterior compartment, as compared to the competing WT winner cells in the anterior compartment (**a**) and comparing the non-competing posterior compartment *RpS3*$^{+/-}$ cells to *RpS3*$^{+/-}$ cells in the centre region of the competing patches in the anterior compartment (**b**). Graphs display three separate experimental replicates, wherein each dot corresponds to an individual wing disc. Statistics reflect 2-sided Wilcoxon signed rank test with Cliff's δ effect size metric. Box and whisker plot denotes minimum, first quartile, median, third quartile, and maximum. Biologically independent samples per replicate are as follows: replicate 1: n=7; replicate 2: n=9; replicate 3: n=11. The scale bars correspond to 50µm. Source data are provided as a Source Data file.
