# Supplementary material for: The PECAn image and statistical analysis pipeline identifies Minute cell competition genes and features
Source: Nat Commun. 2023 May 10;14:2686. doi: 10.1038/s41467-023-38287-x (PMC10172353; doi:10.1038/s41467-023-38287-x)
Supplement: Supplementary file 2 — Reporting Summary [file 41467_2023_38287_MOESM2_ESM.pdf]

nature portfolio

Corresponding author(s): Eugenia Piddini and Michael Baumgartner

Last updated by author(s): Apr 6, 2023

Reporting Summary

Nature Portfolio wishes to improve the reproducibility of the work that we publish. This form provides structure for consistency and transparency in reporting. For further information on Nature Portfolio policies, see our Editorial Policies and the Editorial Policy Checklist.

Statistics

For all statistical analyses, confirm that the following items are present in the figure legend, table legend, main text, or Methods section.

n/a	Confirmed	
☐	☒	The exact sample size (n) for each experimental group/condition, given as a discrete number and unit of measurement
☐	☒	A statement on whether measurements were taken from distinct samples or whether the same sample was measured repeatedly
☐	☒	The statistical test(s) used AND whether they are one- or two-sided<br>*Only common tests should be described solely by name; describe more complex techniques in the Methods section.*
☐	☒	A description of all covariates tested
☐	☒	A description of any assumptions or corrections, such as tests of normality and adjustment for multiple comparisons
☐	☒	A full description of the statistical parameters including central tendency (e.g. means) or other basic estimates (e.g. regression coefficient) AND variation (e.g. standard deviation) or associated estimates of uncertainty (e.g. confidence intervals)
☐	☒	For null hypothesis testing, the test statistic (e.g. F, t, r) with confidence intervals, effect sizes, degrees of freedom and P value noted<br>*Give P values as exact values whenever suitable.*
☒	☐	For Bayesian analysis, information on the choice of priors and Markov chain Monte Carlo settings
☒	☐	For hierarchical and complex designs, identification of the appropriate level for tests and full reporting of outcomes
☐	☒	Estimates of effect sizes (e.g. Cohen's d, Pearson's r), indicating how they were calculated

Our web collection on statistics for biologists contains articles on many of the points above.

Software and code

Policy information about availability of computer code

Data collection	All data used in this paper derives from confocal images of samples prepared within the Piddini Lab. All images were acquired using a Leica SPS confocal microscope combined with LAS AF 2.7.3.9723 software.
Data analysis	Statistical tests comparing manual vs macro output were made using Graphpad Prism 9 software. All further data was generated using the custom PECAn FIJI plugin and associated R shiny app (https://github.com/mebaumgartner/Michaels_Magic_Macro and https://michaelbaumgartner.shinyapps.io/Macro_Analysis_App/) . FIJI 1.53d was used, along with Jython 2.7.2, R 3.6.3 and shiny 1.5.0. The Pipeline for Enhanced Clonal Analysis (PECAn) software was designed in the script editor of ImageJ299 FIJI version 1.53d9. All code was written in the Jython programming language. In addition to base FIJI packages, the Bio-Voxxel, MorphoLibJ11, and the IJ-plugins toolkit were used. The R shiny analysis application was made in RStudio using R3.6.3 and shiny 1.5.0. The analysis app uses the following external libraries: PerformanceAnalytics, ggplot2, boot, MASS, car, RColorBrewer, ggpubr, markdown, ggsignif, rhandsontable, msm, dplyr, magrittr, ICSNP, mvnormtest, psych, corrplot, rcompanion, stringr, effsize, sandwich, ggthemes, shinyBS, reshape2, and effects.

For manuscripts utilizing custom algorithms or software that are central to the research but not yet described in published literature, software must be made available to editors and reviewers. We strongly encourage code deposition in a community repository (e.g. GitHub). See the Nature Portfolio guidelines for submitting code & software for further information.

Data

Policy information about availability of data

All manuscripts must include a data availability statement. This statement should provide the following information, where applicable:

- Accession codes, unique identifiers, or web links for publicly available datasets
- A description of any restrictions on data availability
- For clinical datasets or third party data, please ensure that the statement adheres to our policy

Source data are provided with this paper and are available as a source data file. The RNA-sequencing dataset generated in this study have been deposited in the Gene Expression Omnibus repository with the accession number of GSE181165.

Human research participants

Policy information about studies involving human research participants and Sex and Gender in Research.

Reporting on sex and gender	No human research participants were included in this study. The sexes of all Drosophila samples is specified throughout the manuscript.
Population characteristics	N/A
Recruitment	N/A
Ethics oversight	N/A

Note that full information on the approval of the study protocol must also be provided in the manuscript.

Field-specific reporting

Please select the one below that is the best fit for your research. If you are not sure, read the appropriate sections before making your selection.

[x] Life sciences [ ] Behavioural & social sciences [ ] Ecological, evolutionary & environmental sciences

For a reference copy of the document with all sections, see nature.com/documents/nr-reporting-summary-flat.pdf

Life sciences study design

All studies must disclose on these points even when the disclosure is negative.

Sample size	No calculations were performed to determine an appropriate sample size. Sample sizes were chosen to reflect general practice within the competition field.
Data exclusions	In the screen, any datasets wherein the control group was considered a significant outlier by clone size and/or border death metrics were excluded from the final analysis.
Replication	All datasets with replicates are provided in the accompanying source data file. For novel findings, results were independently replicated at least once. For simple controls to evaluate software performance for which biological variability was not deemed a significant concern, one replicate was deemed sufficient.
Randomization	Data were allocated to experimental groups by genotype
Blinding	Data were not blinded. For tests wherein manual quantifications were compared against software output, the same dataset was analyzed in two separate ways, therefore, blinding was not warranted. For all other experiments, including the screen, all datasets were automatically processed by the software, therefore blinding was not deemed necessary or applicable..

Reporting for specific materials, systems and methods

We require information from authors about some types of materials, experimental systems and methods used in many studies. Here, indicate whether each material, system or method listed is relevant to your study. If you are not sure if a list item applies to your research, read the appropriate section before selecting a response.

Materials & experimental systems

n/a	Involved in the study	
	☒	Antibodies
☒	☐	Eukaryotic cell lines
☒	☐	Palaeontology and archaeology
	☒	Animals and other organisms
☒	☐	Clinical data
☒	☐	Dual use research of concern

Methods

n/a	Involved in the study	
☒	☐	ChIP-seq
☒	☐	Flow cytometry
☒	☐	MRI-based neuroimaging

Antibodies

Antibodies used	Primary antibodies used were: Rabbit anti-DCP-1 antibody (1:2000, Cell Signalling, cat#9578S), Rabbit anti-p-eIF2α (1:500, Cell Signalling, cat#3398T) and mouse anti-beta galactosidase (1:500, Promega, cat#Z3781), rat anti-Ci (1:1000, DSHB 2A1), and rabbit anti-ref(2)P (1:2000, generated by Tor Eric Rusten and published in PMID: 28077876). Secondary antibodies used were donkey anti-rabbit IgG Alexa Fluor 555 (1:500, Thermo Scientific, cat#A31572), donkey anti-rabbit IgG Alexa Fluor 488 (1:500, Thermo Scientific, cat#A21206), goat anti-rat IgG 647 (1:500, Thermo Scientific, A21247), goat anti-mouse IgG 555 (1:500, Thermo Scientific, A21127)
Validation	We have previously published our results with the anti-DCP1, anti-ref(2)P, anti-p-eIF2α, anti-beta glactosidase, and anti-Ci antibodies (PMID: 33495633, PMID: 34914692, PMID: 28743877) The anti-DCP1 and anti-peIF2α antibodies are endorsed for IF usage by the manufacturer and are well cited in the literature (e.g. PMID: 35179490). Results in our hands are furthermore consistent with this: conditions reported to suppress apoptosis in competing wing discs (Xrpl-RNAi, Dronc-RNAi) indeed show a decrease in apoptosis in our system using this antibody (See figure 2g-h). The anti-Ref(2)P was generated and described previously (PMID: 28077876). For all secondary antibodies, advanced verification statements are available on Thermo Fisher's website.

Animals and other research organisms

Policy information about studies involving animals; ARRIVE guidelines recommended for reporting animal research, and Sex and Gender in Research

Laboratory animals	Unless otherwise specified, all samples were dissected at the wandering third instar stage, 6 days after egg laying. An extensive list of Drosophila lines were used. The sources are detailed in the methods section, and the lines used in the screen are provided in supplementary_data_6.
Wild animals	No wild animals were used
Reporting on sex	The sexes of Drosophila samples is indicated throughout. Unless otherwise stated, the sexes of all samples were females.
Field-collected samples	No field-collected samples were used
Ethics oversight	No ethical approval was needed, as the study used exclusively Drosophila or other archival datasets.

Note that full information on the approval of the study protocol must also be provided in the manuscript.
