# Supplementary material for: The PECAn image and statistical analysis pipeline identifies Minute cell competition genes and features
Source: Nat Commun. 2023 May 10;14:2686. doi: 10.1038/s41467-023-38287-x (PMC10172353; doi:10.1038/s41467-023-38287-x)
Supplement: Supplementary file 3 — Description of Additional Supplementary Files [file 41467_2023_38287_MOESM3_ESM.pdf]

Title: Supplementary Data 1
Description: Validation of PECAn multivariate regression tools

Title: Supplementary Data 2
Description: Results of single cell logistic regression analysis

Title: Supplementary Data 3
Description: Results of population-level logistic regression analysis

Title: Supplementary Data 4
Description: RNA-seq results for identifying Nrf2/loser candidate targets for screen

Title: Supplementary Data 5
Description: List of all target genes identified for screen

Title: Supplementary Data 6
Description: Results of RNAi screen listed by RNAi line used

Title: Supplementary Data 7
Description: Experimental genotypes and clone induction conditions

Title: Supplementary Video 1
Description: Example of PECAn image analysis output image for competition experiment
